# Flavivirus-Mediating B Cell Differentiation Into Antibody-Secreting Cells in Humans Is Associated With the Activation of the Tryptophan Metabolism

**DOI:** 10.3389/fimmu.2020.00020

**Published:** 2020-02-11

**Authors:** Vivian Bonezi, Allan H. D. Cataneo, Maryana S. F. Branquinho, Maysa B. B. Silva, Patricia Gonzalez-Dias, Samuel S. Pereira, Luís C. de Souza Ferreira, Helder I. Nakaya, Ana Campa, Pryscilla F. Wowk, Eduardo L. V. Silveira

**Affiliations:** ^1^Department of Clinical and Toxicological Analyses, School of Pharmaceutical Sciences, University of São Paulo, São Paulo, Brazil; ^2^Laboratório de Virologia Molecular, Instituto Carlos Chagas (ICC/Fiocruz Paraná), Curitiba, Brazil; ^3^Scientific Platform Pasteur, University of São Paulo, São Paulo, Brazil; ^4^Department of Microbiology, Institute of Biomedical Sciences, University of São Paulo, São Paulo, Brazil

**Keywords:** Dengue virus, B cell differentiation, antibody-secreting cells, tryptophan metabolism, flaviviruses

## Abstract

Patients infected with the Dengue virus (DENV) often present with a massive generation of DENV-specific antibody-secreting cells (ASCs) in the blood. In some cases, these ASCs represent more than 50% of the circulating B cells, a higher magnitude than those induced by other infections, vaccinations, and plasma cell lymphomas. However, it remains unclear how the DENV infection elicits this colossal response. To address this issue, we utilised an *in vitro* strategy to induce human PBMCs of healthy individuals incubated with DENV particles (DENV4 TVP/360) to differentiate into ASCs. As controls, PBMCs were incubated with a mitogen cocktail or supernatants of uninfected C6/36 cells (mock). The ASC phenotype and function were increasingly detected in the DENV and mitogen-cultured PBMCs as compared to mock-treated cells. In contrast to the *in vivo* condition, secreted IgG derived from the PBMC-DENV culture was not DENV-specific. Lower ASC numbers were observed when inactivated viral particles or purified B cells were added to the cultures. The physical contact was essential between B cells and the remaining PBMCs for the DENV-mediated ASC response. Considering the evidence for the activation of the tryptophan metabolism detected in the serum of Dengue patients, we assessed its relevance in the DENV-mediated ASC differentiation. For this, tryptophan and its respective metabolites were quantified in the supernatants of cell cultures through mass spectrophotometry. Tryptophan depletion and kynurenine accumulation were found in the supernatants of PBMC-DENV cultures, which presented enhanced detection of indoleamine 2,3-dioxygenase 1 and 2 transcripts as compared to controls. In PBMC-DENV cultures, tryptophan and kynurenine levels strongly correlated to the respective ASC numbers, while the kynurenine levels were directly proportional to the secreted IgG titers. Contrastingly, PBMCs incubated with Zika or attenuated Yellow Fever viruses showed no correlation between their kynurenine concentrations and ASC numbers. Therefore, our data revealed the existence of distinct pathways for the DENV-mediated ASC differentiation and suggest the involvement of the tryptophan metabolism in this cellular process triggered by flavivirus infections.

## Introduction

Dengue is an acute infectious disease caused by Dengue virus (DENV) transmission through mosquito bites (*Aedes aegypti* or *Aedes albopictus*). This flavivirus-related disease has been widespread in tropical countries. Its global burden reaches nearly eight billion dollars/year due to the morbidity and mortality derived from almost 400 million infections. Moreover, 40% of the world population remains at risk for that viral infection (1). All four DENV serotypes (1–4) can similarly infect and elicit pathophysiology in humans. Regarding the clinical forms of the disease, DENV-infected individuals can be anything from asymptomatic to displaying mild or even severe symptoms that range from hemorrhage or shock syndrome to death (2).

This viral infection triggers robust innate and adaptive immune responses. About the innate responses, DENV particles stimulate the interferon (IFN) production (3, 4) upon the cell invasion and replication that occurs in multiple cell subsets, including monocytes as well as T and B lymphocytes (5). The augmented expression of IFN-stimulated genes ends up activating multiple cell signalling pathways in several cell subsets. Among them, the IFN-gamma interference is distinguished in the activity of some metabolic enzymes, such as the indoleamine 2,3-dioxygenase (IDO) (6). During the DENV infection, IDO promotes the tryptophan degradation, resulting in the accumulation of kynurenine and subsequent metabolites in the patient serum concomitantly with the symptomatology (7). Regarding the adaptive responses, T and B cells are activated upon DENV infection, leading to the further cell differentiation and secretion of DENV-specific neutralizing antibodies (nAbs). Despite the potent nAbs generated against the primary DENV serotype infection, some of them are quite effective against a different viral serotype from a secondary infection (8–10).

Intriguingly, the DENV infection triggers a substantial and transient antibody-secreting cell (ASC) response simultaneously to the symptomatology. Those DENV infection-elicited ASCs are highly specific to viral envelope epitopes and represent more than 50% of all circulating B cells on average. That percentage can still rise if a more severe DENV infection or a secondary viral exposure with a distinct serotype is considered (11, 12). Quantitatively, this DENV-specific ASC response presents a much higher magnitude than the counterparts induced by viral infections (Ebola, Andes, Influenza) (13–15), immunisations (attenuated Yellow Fever virus 17DD or inactivated Influenza vaccine) (11–16), and plasma cell lymphoma (17).

In order to elucidate the underlying requirements behind this massive DENV-induced ASC response seen in humans, we initially developed an *in vitro* assay based on the culture of PBMCs from healthy individuals. Then, we investigated the influence of certain parameters over the B cell ability to acquire the ASC phenotype and function: (a) viable vs. inactivated DENV particles; (b) PBMCs vs. purified CD19+ B cells; and (c) lack of cell-cell contact between purified CD19+ B cells and remaining PBMCs. Also, we evaluated whether those PBMC cultures with DENV or flaviviruses, such as Zika and Yellow Fever, had the tryptophan metabolism activated and whether they correlated with their respective ASC generation and IgG secretion.

## Materials and Methods

### Viruses, Blood Samples, and Cell Cultures

All flavivirus strains used in this study are part of the viral collection CVAM from the Laboratório de Virologia Molecular of the Instituto Carlos Chagas/Fiocruz-PR (ICC/Fiocruz-PR) (Brazil). Among them, we tested Dengue viruses [the lab-adapted DENV4 TVP/ 360 and clinical isolate DENV4 LRV13/422 (Genbank accession number KU513442 and KU513441, respectively)], Zika viruses [an African lineage (ZIKV-MR766) (18) and ZIKV-PE243, a Brazilian isolate from Asian lineage] (19), and the attenuated Yellow Fever virus used for vaccination (YFV 17DD) (20). All the DENV and ZIKV strains used in this study were previously expanded and isolated from C6/36 cells (*A. albopictus* mosquitoes) except for the attenuated yellow fever virus strain 17DD particles that were grown in Vero cell cultures.

Eight to ten millilitres of peripheral blood were collected from healthy adult individuals at the University of São Paulo and Instituto Carlos Chagas, Fiocruz/PR, Brazil. After the Ficoll gradient isolation, PBMCs were cultivated in different conditions to have their B cells differentiated into antibody-secreting cells (ASCs). For this, PBMCs were incubated at 37°C in a 5% CO_2_ incubator with individual flaviviruses with a multiplicity of infection (MOI) of 10 for 7 days (5). As controls, PBMCs were cultivated with the supernatant of uninfected C6/36 cells (Mock) or a mitogen cocktail [Pokeweed mitogen (PWM), *Streptococcus aureus* cowan (SAC), CpG ODN 2006 and β-mercaptoethanol] as previously described (21). To have their potential to differentiate into ASCs, CD19+ B cells were isolated from PBMCs through positive selection with magnetic beads (Miltenyi) before being stimulated. All experimental protocols and procedures were reviewed and approved by the Ethics Committee regulated by the Conselho Nacional de Ética em Pesquisa (Process No. 68875417.9.0000.0067).

### Flow Cytometry

The cell staining for the ASC phenotype (CD20neg CD27hi CD38hi) was performed on the seventh day of culture with harvested cells using the described antibodies (Supplementary Table 1). Cell staining was performed for 30 min at 4°C in the dark. After two washing steps, the cell acquisition was done in a BD FACSCanto™ II from the PDTIS flow cytometry facility (ICC/Fiocruz-PR). The details of these analyses were evaluated with the FlowJo software.

### B Cell ELISPOT

The enumeration of IgG-secreting cells was performed on day 7 of those cultures as previously described (22). A heatmap was designed based on Z-scores. Due to the wide range of spot enumeration per sample, those values were transformed into a normalised basic unit of comparison called the Z-score. Positive values indicated that the data were above average, and negative values were below average.

### Mass Spectrometry

The supernatants of cell cultures from day 7 were gathered (nearly 300 μL) and analysed for the presence of tryptophan-derived metabolites. For this, they were initially incubated with 700 μL of a 0.1% acetic acid solution containing methanol:acetone (1:1). Then, 10 μL of a standard mix solution (MLT-D4 e Trp-D5) was added before homogenisation with a vortex for 1 min. The homogenised solution was stored for 30 min at −20°C for protein precipitation. A 14,000 × g centrifugation was applied to the tubes for 10 min at 4°C, and the supernatants were transferred to new tubes under the nitrogen gas atmosphere for drying. Samples were reconstituted in 100 μL of a solution containing water:methanol (9:1), and the injection volume corresponded to 2 μL applied at a flow rate of 200 μL/min into a LC-MS/MS [LC 1250 Bin Pump VL and 1260 HiP ALS autosampler coupled to a triple quadrupole 6460 mass spectrometer (Agilent Technologies, CA, USA)]. The dependence linearity of the concentration response was verified by regression analysis. The electrospray ion source (ESI) was operated in the positive mode. MS data were acquired by multiple reaction monitoring (MRM) mode and evaluated through the MassHunter Quantitative Analysis software (Agilent Technologies). A Luna C18 [150 mm × 2 mm, 3 μm] reversed-phase column (Phenomenex, CA, USA) was used for the LC separation. Chromatography was performed through a gradient elution composed by water, 0.5% formic acid and 2 mM ammonium formate in an mix of acetonitrile:water (9:1) (adjusted with 0.5% formic acid). Data normalisation for each metabolite were performed by subtracting the sample values from those obtained with culture medium kept under the same conditions without cells.

### cDNA Synthesis and Quantitative Real Time PCR

The gene expression analyses were measured through quantitative real time PCR (qRT-PCR) with a 7500 FAST Real Time System thermocycler™ (Thermo Fisher Scientific) as described below. Briefly, 2 μg of total RNA of those cell cultures were used as templates for the cDNA synthesis. For this, we used the *SuperScript Vilo Master Mix*™ (Life Technologies) and ultra-pure water as recommended by the manufacturer. The reaction was run in a thermocycler with adjusted settings: 65°C for 5 min, 50°C for 120 min, and 95°C for 5 min. cDNA samples were stored in a −20°C freezer until the use.

Different genes potentially related to the ASC differentiation were targeted (*SYK, IL-10, SRC, TNFS13B, IDO1*, and *IDO2*) with those cDNAs. Those targets were amplified with the SYBR™ Green Master Mix (Thermo Fisher Scientific) and the following primers (Supplementary Table 2). The PCR settings were 50°C for 2 min, 95°C for 10 min, 45 cycles of 95°C for 15 s, and 60°C for 1 min. The relative gene expression (ΔCt) was calculated by the difference between the cycle threshold (Ct) of the target gene and the Ct of the reference gene. The fold change data were obtained through the formula 2^∧^-ΔΔCt = (ΔCt of treated group – ΔCt of the control group) (23), using the Mock conditions as the control group.

The presence of intracellular viral particles was also estimated from the cell cultures through qRT-PCR. DENV-specific primers (Supplementary Table 2) were used to amplify those cDNAs with the SYBR™ Green Master Mix. For this, the PCR settings were 50°C for 2 min, 95°C for 10 min, 45 cycles of 95°C for 15 s, and 67°C for 1 min.

### ELISA

Plasma samples derived from the healthy individuals were assessed for the presence of anti-DENV1-4 IgG antibodies through ELISA assays against NS1 and EDIII recombinant proteins and viral particles. Briefly, ELISA plates were coated with a pool of recombinant proteins (DENV1-4 rNS1 or EDIII - 550 ng/well) or viral particles (DENV1-4 - 200 ng/well) in carbonate buffer (pH 9.6) overnight at 4°C. After four washing steps with a 0.5% Tween 20-PBS solution, the plate wells were blocked for 2 h at 37°C with a 3% skimmed milk solution containing 0.5% fetal bovine serum (FBS) in a 1X PBS–Tween 0.05% solution. Serum samples were further diluted 1:100 and added to the plates in a serial dilution of 2. After a washing step with a 1X PBS–Tween 0.05% solution, a goat anti-IgG human secondary antibody was added to the plates in a 1:4,000 dilution, which incubation lasts 2 h. Revelation of the assay was performed with a specific buffer (citrate phosphate buffer (pH 5.8), o-Phenylenediamine and hydrogen peroxide) for 15 min in the dark upon another washing step. The revelation was stopped with 2N sulphuric acid (H_2_SO_4_), and the absorbance was measured at 492 nm in an ELISA plate reader (Epoch).

### Flavivirus Titration

Viral titers of DENV4 TVP and ZIKV PE243 were determined in the supernatants of cell cultures on day 7. Briefly, 100 μL of frozen supernatants were thawed and serially diluted 6-fold in Leibovitz's medium 15 (L-15) without FBS and supplemented with 0.26% of tryptose and 25 μg/mL gentamicin. Each dilution was inoculated into C6/36 cells (10^5^ cells/well in 24-well-plates) in duplicate in a well-containing 10^5^ cells and the culture was kept at 28°C for 90 min for the virus adsorption. Then, the inoculum was removed out from the plate and 0.5 mL of CMC overlay medium (L-15 supplemented with 5% SFB, 0.26% tryptose, 25 μg/mL gentamicin, 1.6% carboxymethylcellulose) was added in each plate well. Plates were sealed and incubated for 7 days at 28°C. Then, cells were washed thrice with 1X PBS and fixed with 3% paraformaldehyde solution upon incubation for 20 min. After three washing steps with PBS 1X, cells were permeabilised with 0.5% Triton X-100 solution for 4 min. Cells were washed thrice again with 1X PBS and stained with the anti-envelope protein of flaviviruses 4G2 antibody (ATCC® HB-112™) and diluted 1:200 at 37°C for 60 min. Plates were washed with 1X PBS thrice before their incubation with the anti-mouse alkaline phosphatase (AP) secondary antibody (Promega, Madison, WI, USA) and diluted 1:7,500 at 37°C for 60 min. After the last washing step with 1X PBS, focus forming units (FFUs) were detected by adding a solution of NBT/BCIP (Promega, Madison, WI, USA) as a substrate. Foci were counted and expressed as FFU C6/36/ml.

### Statistical Analyses

These analyses were performed with the software GraphPad Prism for Windows, version 6.0 (GraphPad Software, Inc., La Jolla, CA, EUA). The parametric quantitative variables were compared through a *T*-test or One-way ANOVA with multiple comparisons through the Bonferroni test. The non-parametric quantitative variables were compared through the Mann-Whitney or by Kruskal-Wallis tests with multiple comparisons through the Dunn's test. A *p*-value (*p* < 0.05) represented a significant difference among two groups.

## Results

### DENV Particles Promote the Acquisition of the ASC Phenotype and Function by Human PBMCs *in vitro*

**Figure 1:**
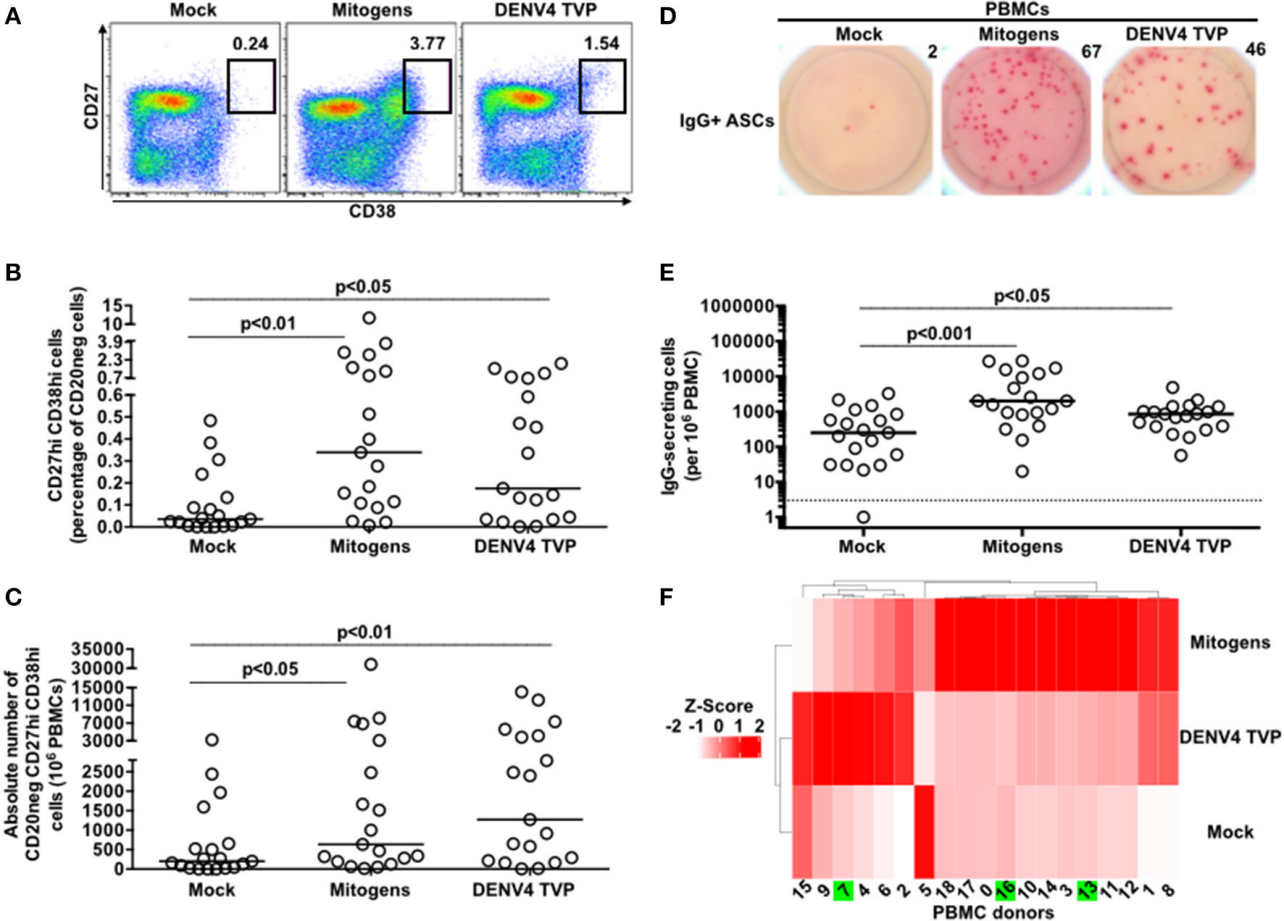
Dengue virus promotes the B cell differentiation into antibody-secreting cells (ASCs) *in vitro* disregarding pre-existing DENV immunity. **(A)** Representative FACS data displaying the percentage of cells with the ASC phenotype at day 7 of culture. **(B,C)** Percentage and absolute number of ASCs quantified through FACS for different PBMC cultures derived from 19 healthy donors. **(D)** Representative data obtained from 3.3 × 10^4^ PBMCs harvested at day 7 of culture and added per well of ELISPOT plates to enumerate IgG-secreting cells in each culture condition. **(E)** Magnitude of IgG-secreting cells detected per 10^6^ PBMCs through ELISPOT. **(F)** Heatmap based on Z scores derived from ELISPOT data for each culture condition. Green boxes represent the PBMC donors that presented pre-existing immunity to DENV.

To estimate the ability of DENV particles to elicit the massive B cell differentiation into ASCs, as seen with human infections, we quantified the ASC phenotype and function in human PBMCs cultivated with the DENV4 TVP strain (TVP/360), a laboratory-adapted reference strain (24). For this, PBMCs derived from 19 healthy individuals were incubated for 7 days with DENV4 TVP particles at MOI of 10. As controls, PBMCs were cultivated with the supernatant of uninfected C6/36 cells (Mock) or a cocktail of Mitogens (CpG ODN, Pokeweed mitogen, and *Staphylococcus aureus* cowan). For the ASC phenotype analysis, we assessed the CD20neg CD27hi CD38hi cell phenotype in the cultures through FACS (Supplementary Figure 1 and Supplementary Table 1). A significant increase in the frequency of cells with the ASC phenotype was detected upon cell culture either with DENV4 TVP (*p* < 0.05; Median and St Dev – 0.175 ± 0.558%) or Mitogens (*p* < 0.01; Median and St Dev – 0.339 ± 2.731%) in comparison to the mock (Median and St Dev – 0.036 ± 0.144%) (Figures 1A,B). In terms of absolute number of ASCs detected per 10^6^ PBMCs, both DENV4 TVP (*p* < 0.01; Median and St Dev – 1,272 ± 4,099) or Mitogen cultures (*p* < 0.05; Median and St Dev – 635 ± 7,170) presented an increased ASC number in comparison to the mock-treated cells (Median and St Dev – 200 ± 948) (Figure 1C). Subsequently, we evaluated whether the incubation with DENV4 TVP prompted the ASC function (i.e., antibody secretion) in those cells through ELISPOT. A significant augment in the frequency of IgG-secreting cells was enumerated with either the DENV4 TVP (*p* < 0.05; Median and St Dev – 853 ± 1,069%) or Mitogens (*p* < 0.001; Median and St Dev – 2,100 ± 9,014) in relation to the mock-treated culture (Median and St Dev – 250 ± 866) (Figures 1D,E). Similar data were also obtained when IgM-secreting ASCs were counted (Supplementary Figure 2). Regarding the IgG secretion, PBMCs cultured with DENV particles were previously shown to present IgG in the supernatants at day 12 of the culture (25). Our data showed that total IgG could be found already at day 7 of the culture (Supplementary Figure 3A). However, all the detected IgG seem to be unspecific to DENV particles (Supplementary Figures 3B,C).

Since two different stimuli (DENV4 TVP and Mitogens) were capable of promoting the ASC phenotype and function, we asked whether such activation could be indiscriminately elicited by themselves. For this, we approached the ELISPOT numbers for IgG-secreting cells that represented not the real cell frequency but the variations among the different stimuli for each sample (Z-score). Interestingly, a group of six samples (#2, 4, 6, 7, 9, and 15) clearly presented a trend to respond better to DENV4 TVP rather than to Mitogens. In contrast, the 13 remaining samples displayed the opposite behaviour (Figure 1F). To verify whether the DENV-responsive samples in the ELISPOT assay were derived from subjects previously infected with DENV, ELISAs were performed to quantify plasma IgG specific to DENV1-4 NS1 and EDIII recombinant proteins and viable DENV particles. For this, plasma samples derived from 13 out of 19 individuals evaluated in this study were used. Although some samples displayed higher optical densities (OD) than the negative control sample against the three different DENV targets, none of them presented OD values as high as the positive control sample did (Supplementary Figures 4A–C). Thus, these data rule out the participation of DENV-specific memory B cells driving the ASC differentiation in this *in vitro* model.

### Inactivated DENV Particles Do Not Activate the ASC Differentiation by Human PBMCs

**Figure 2:**
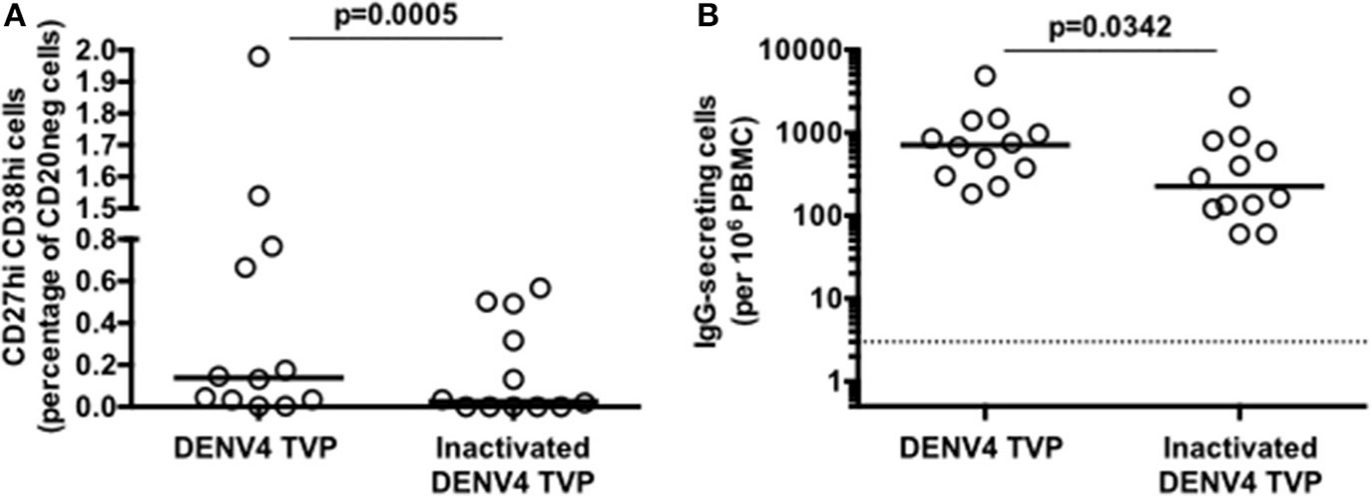
Inactivated Dengue virus particles do not have the same ability to trigger the B cell differentiation into antibody-secreting cells (ASCs) as whole particles do. **(A)** Percentage of ASCs quantified through FACS and **(B)** magnitude of IgG-secreting cells detected through ELISPOT at day 7 of PBMC cultures derived from 12 healthy donors.

To address whether the DENV particle integrity had a role in the B cell differentiation *in vitro*, we cultivated PBMCs with either whole or inactivated DENV4 TVP (UV-treated) particles for 7 days. PBMCs lost the ability to induce ASCs either phenotypically upon the incubation with inactivated viral particles [*p* < 0.0005 – Whole (Median and St Dev – 0.14 ± 0.66) vs. Inactivated particles (Median and St Dev – 0.03 ± 0.23)] and functionally [*p* < 0.0342 – Whole (Median and St Dev – 712 ± 1,274) vs. Inactivated particles (Median and St Dev – 225 ± 742)] (Figures 2A,B, respectively). The ASC generation provided by this model of cell culture can therefore be observed only with whole DENV particles.

### Viable DENV Particles Are Unable to Trigger the ASC Function in Purified CD19^+^ B Cell Culture

**Figure 3:**
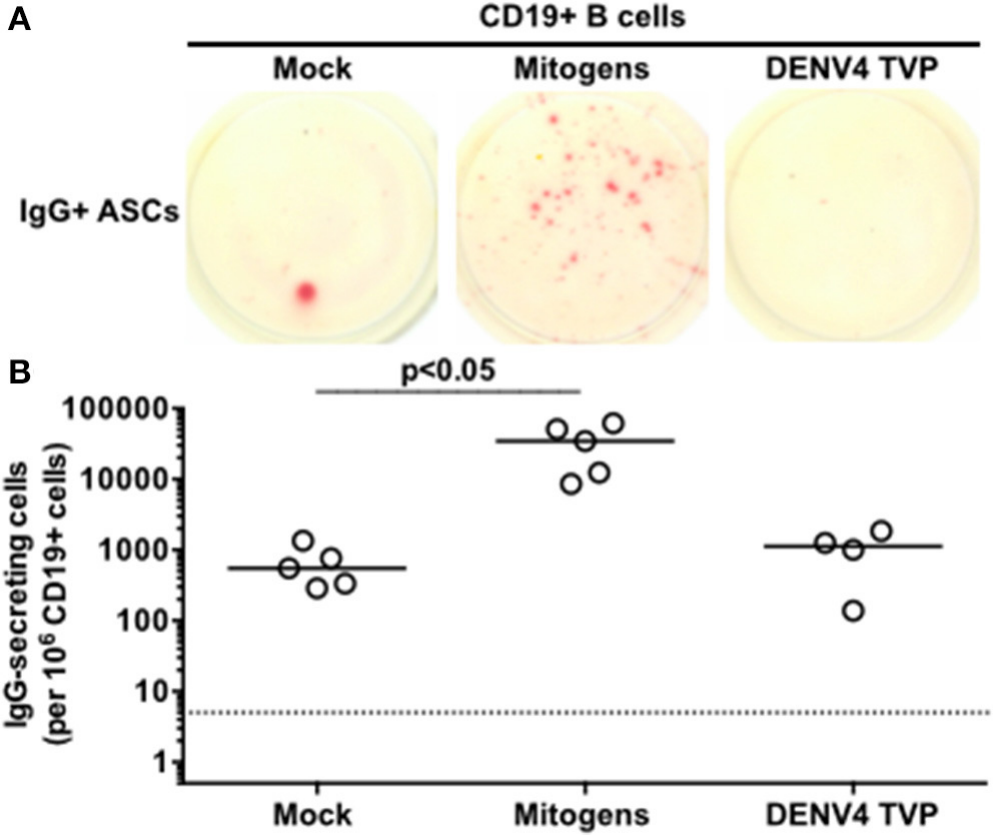
Dengue virus does not trigger the ASC differentiation when incubated with isolated B cells. CD19+ B cells were isolated from PBMCs of six donors and five of them were tested in this assay, except for DENV4 TVP incubation (*n* = 4 samples). **(A)** Representative data obtained from 6.6 × 10^4^ CD19+ B cells harvested at day 7 of culture and added per well of ELISPOT plates to enumerate IgG-secreting cells in each culture condition. **(B)** Magnitude of IgG-secreting cells detected per 10^6^ CD19+ B cells through ELISPOT.

Since only viable DENV4 TVP particles could stimulate the acquisition of the ASC phenotype and function in human PBMCs *in vitro*, can these viral particles reproduce the same cell differentiation in purified B cells? CD19^+^ B cells were isolated from the same tested PBMCs with anti-human CD19-specific magnetic beads and cultivated with viable viral particles for the enumeration of IgG-secreting cells at day 7. Meanwhile, Mitogens induced a significant CD19+ B cell differentiation into ASCs in comparison to Mock-treated cells [*p* < 0.05; Mitogens (Median and St Dev – 34,560 ± 23,174) vs. Mock (Median and St Dev – 550 ± 431)]. DENV4 TVP particles showed no significant activation of this process [*p* > 0.05; DENV4 TVP (Median and St Dev – 1,125 ± 710)] (Figures 3A,B). Hence, we suggest that the ASC differentiation is dependent on either physical cell contact or the secretion of soluble B cell stimulator by the remaining PBMCs in PBMCs cultivated with DENV4 TVP.

### DENV-Mediated ASC Differentiation Is Dependent on the B Cell-Remaining PBMC Contact

**Figure 4:**
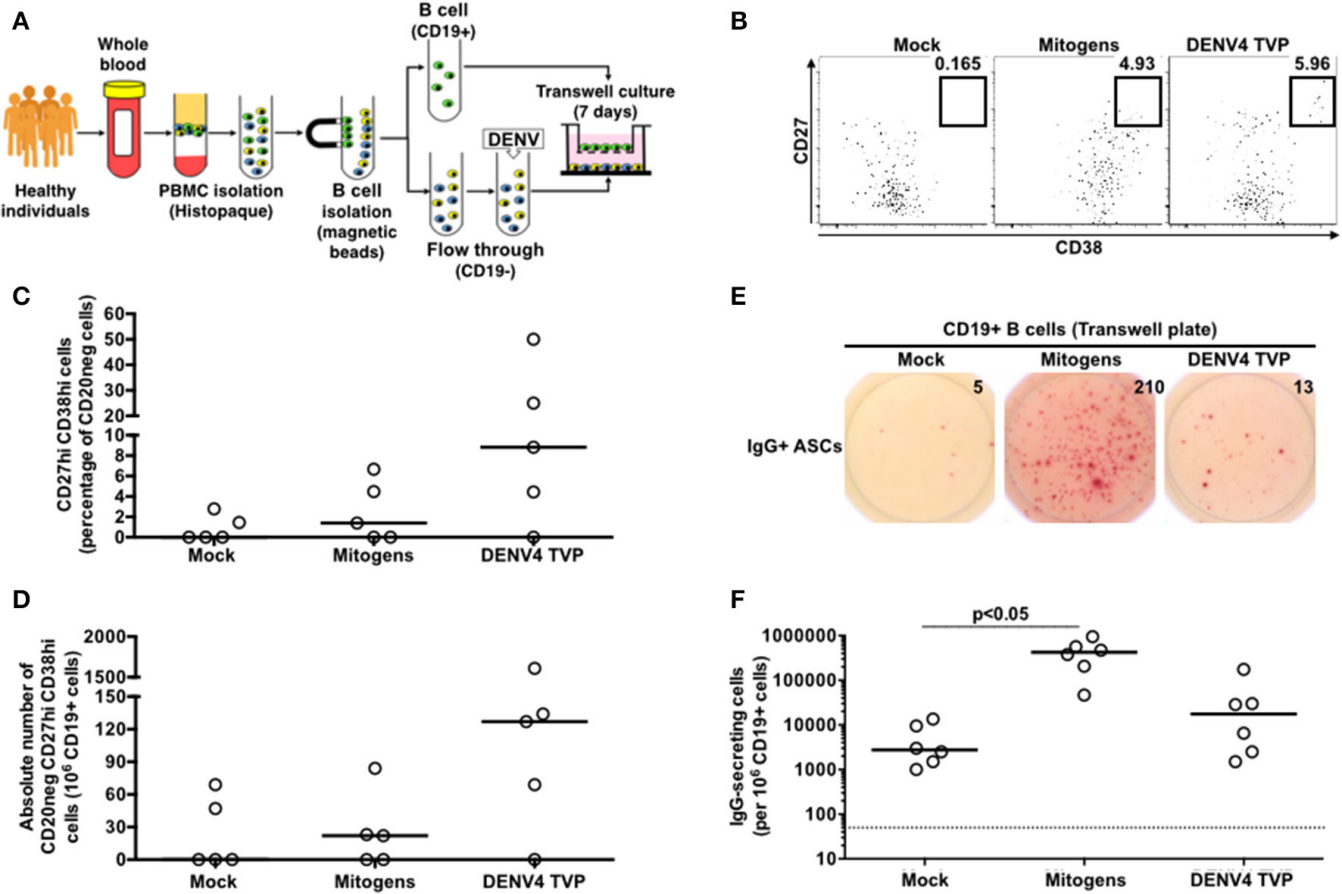
DENV-mediated ASC differentiation is dependent on the physical contact between CD19+ B cells and the remaining PBMCs. **(A)** CD19+ B cells were isolated from PBMCs (*n* = 5) and cultured without physical contact with the flow through (CD19– cells) (in a 1:10 ratio, respectively) in transwell plates. CD19+ B cells were harvested at day 7 of culture and had the ASC phenotype **(B–D)** and function **(E,F)** assessed. **(F)** A total of 2.2 × 10^4^ CD19+ cells were added per well, except for the Mitogen culture (2.2 × 10^3^ CD19+ cells).

To investigate whether the cell physical contact was critical for the DENV4 TVP-mediated ASC differentiation detected *in vitro*, we cultivated purified CD19^+^ B cells with the remaining PBMCs infected with DENV4 TVP (in a 1:10 ratio respectively) in a transwell plate. CD19+ B cells were harvested on day 7 of culture and probed for the ASC phenotype and function. Although DENV4 TVP particles elicited higher ASC responses in the culture, measured by the cell percentage (Median and St Dev – 8.82 ± 20.4%) and absolute numbers of CD20neg CD27hi CD38hi cells (Median and St Dev – 127 ± 684), those numbers did not differ from the mock condition (ASC %: Median and St Dev – 0 ± 1.25%; ASC absolute number: Median and St Dev – 0 ± 33). Moreover, Mitogens were not able to significantly increase the ASC frequency (ASC %: Median and St Dev – 1.4 ± 3.0%; ASC absolute number: Median and St Dev – 22 ± 34) (Figures 4A–D). Furthermore, whereas DENV4 TVP generated similar IgG+ ASC numbers (Median and St Dev – 17.5 × 10^3^ ± 67.2 × 10^3^) in comparison to mock (2.75 × 10^3^ ± 5.11 × 10^3^), Mitogens elicited a massive IgG+ ASC differentiation in the transwell culture (*p* < 0.05 – Median and St Dev – 425 × 10^3^ ± 311 × 10^3^) (Figures 4E,F). To fully differentiate into ASCs in our model, CD19^+^ B cells must have the physical contact with the DENV4 TVP exposed-remaining PBMCs.

### Tryptophan Metabolism Distinguishes the ASC Differentiation Mediated by DENV and Mitogens *in vitro*

**Figure 5:**
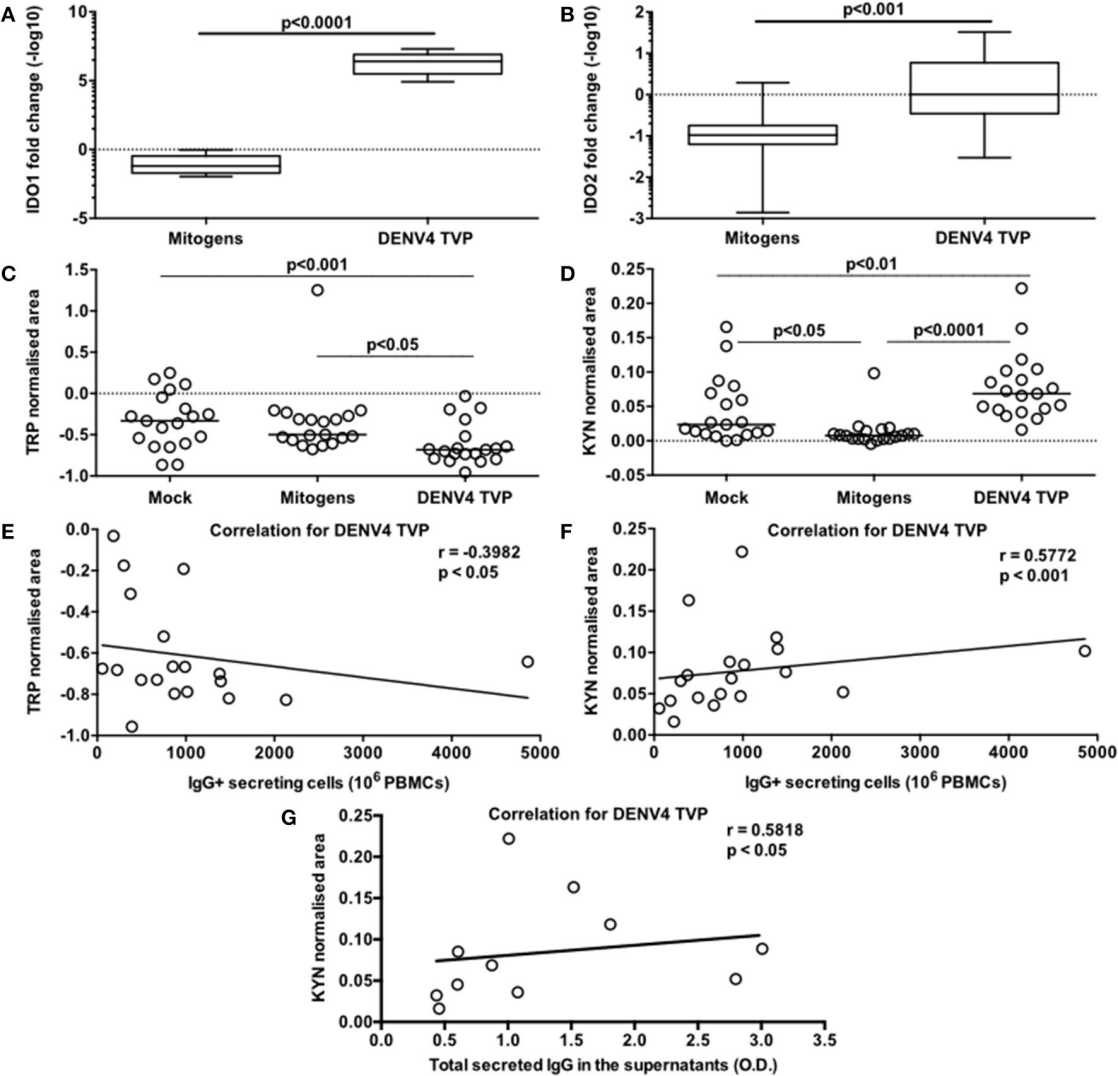
Activation of the tryptophan metabolism is strongly associated with the DENV-mediated ASC differentiation detected in PBMCs *in vitro*. Different PBMC cultures derived from 19 healthy donors had their cells and supernatants harvested at day 7 of the culture. To verify the participation of the tryptophan metabolism in this cellular process, the following parameters were measured: **(A,B)** gene expression levels of *IDO1* and *IDO2* respectively in the cells; **(C)** Tryptophan (Trp) consumption and **(D)** Kynurenine (Kyn) accumulation in the supernatants; **(E)** correlation between the Trp consumption and ASC numbers (counted through ELISPOT); correlations between the Kyn accumulation and ASC numbers **(F)** or the total amount of secreted IgG in the supernatants **(G)**. **(A,B)** Data are represented as boxes and whiskers (10–90% percentile). Dashed line represents the gene expression within the Mock culture condition. **(C,D)** Dashed lines represent the standard amount of the metabolite found in the culture medium kept under the same conditions, but without cells.

DENV4 TVP and Mitogens clearly induced phenotypical and functional ASC differentiation when incubated with PBMCs. Is this cellular process regulated by the same metabolic pathway for both stimulators? To answer this question, we initially investigated the transcriptome of those PBMC cultures through qRT-PCR. As Dengue patients seem to present with lower tryptophan and higher kynurenine concentrations in the serum (7), we decided to include *IDO1* and *IDO2* genes in the analysis. Surprisingly, we observed increased amounts for both IDO transcripts in the PBMC-DENV4 TVP culture (*p* < 0.0001 – *IDO1* – Median and St Dev – 2.5 × 10^6^ ± 6.6 × 10^6^; *p* < 0.001 – IDO2 – Median and St Dev – 1.01 ± 13.07) in comparison to the Mitogen counterpart (*IDO1* – Median and St Dev – 0.065 ± 0.296; *IDO2* – Median and St Dev – 0.105 ± 0.668) (Figures 5A,B, respectively). Other targets associated to the cellular signalling (*SYK* and *SRC*) and the B cell differentiation into ASCs (*TNFS13B* and *IL-10*) also had their expression evaluated. For all of them (*TNFS13B, IL-10, SYK*, and *SRC*), the DENV4 TVP-stimulated PBMCs displayed increased transcript amounts with regard to Mitogen-exposed cells (Supplementary Figures 5A–D). Although either DENV4 TVP and Mitogens were capable of fully differentiating PBMC-containing CD19^+^ B cells into ASCs, our qRT-PCR data suggest that each stimulator could elicit different metabolic and signalling pathways during this *in vitro* process.

Considering the data paucity regarding the metabolism influence on the B cell response and higher concentrations of serum kynurenine found in Dengue patients (7), we also assessed the participation of the tryptophan metabolism in our model. For this, we measured the normalised areas occupied by the tryptophan and kynurenine peaks obtained through mass spectrometry in the culture supernatants at day 7. The PBMCs cultured with DENV4 TVP showed the highest tryptophan consumption (Median and St Dev: 0.682 ± 0.252) in comparison to the Mock (*p* < 0.001 – Median and St Dev: −0.333 ± 0.332) or Mitogen cultures (*p* < 0.05 – Median and St Dev: −0.499 ± 0.419) (Figure 5C). Moreover, the supernatants from the PBMC-DENV4 TVP culture had the highest kynurenine concentration (Median and St Dev – 0.07 ± 0.05) in relation to the Mock (*p* < 0.01 – Median and St Dev – 0.024 ± 0.047) or Mitogen counterparts (*p* < 0.0001 – Median and St Dev – 0.008 ± 0.022) (Figure 5D). Notably, the tryptophan degradation and kynurenine accumulation detected in the DENV4 TVP-treated cultures presented correlations with the magnitude of the IgG+ ASCs enumerated at day 7 (respectively *r* = −0.3982; *p* < 0.05; *r* = 0.5772; *p* < 0.001) (Figures 5E,F) and the titers of secreted IgG (*r* = 0.5818; *p* < 0.05) (Figure 5G). Other metabolites downstream of this metabolic pathway (5-hydroxyindoleacetic, anthranilic, kynurenic, quinolinic, and nicotinic acids) were also detected in higher amounts for DENV4 TVP in comparison to Mitogen cultures (Supplementary Figures 6A–E).

### Activation of the Tryptophan Metabolism Portrays the Flavivirus-Mediated ASC Response *in vitro*

**Figure 6:**
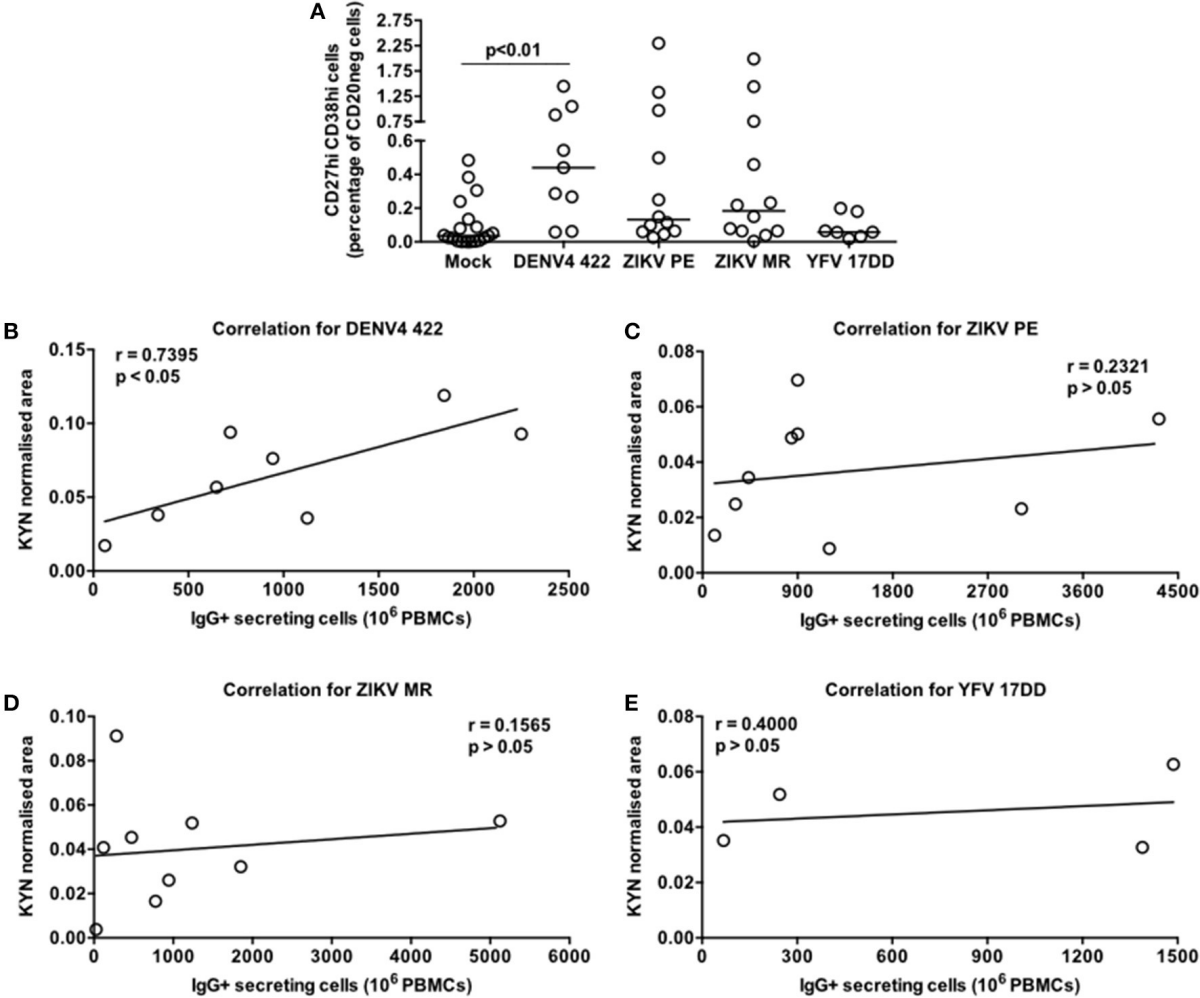
Contrasting pattern of the tryptophan metabolism induced in PBMC cultures stimulated by a DENV4 clinical isolate (422) or flaviviruses that elicit low ASC responses *in vivo* (ZIKV MR766, ZIKV PE243, and attenuated Yellow Fever 17DD). Different PBMC cultures derived from healthy donors had cells and supernatants obtained at day 7 of the cultures. **(A)** ASC percentage quantified through FACS for different flavivirus-stimulated PBMC cultures. **(B–E)** Correlation between the Kyn levels and ASC numbers (counted through ELISPOT) derived from each cell culture.

Since the DENV-induced ASC differentiation described herein was performed with a laboratory adapted reference viral strain we sought to determine whether the exposure to a clinical DENV isolate and other flaviviruses could elicit similar responses. Then, we selected the DENV4 LRV13/422 (24) as well as two Zika virus strains (ZIKV PE243 and ZIKV MR766) and the Yellow fever virus vaccine strain (YFV 17DD) for further analysis. We detected a significant augment of CD20neg CD27hi CD38hi cells only in the PBMC-DENV4 LRV13/422 culture in comparison to mock (*p* < 0.01 – DENV4 LRV13/422 – Median and St Dev – 0.44 ± 0.476; Mock – Median and St Dev – 0.036 ± 0.144). The remaining flaviviruses showed similar ASC percentages to mock (Figure 6A), whereas DENV infections have been associated with a massive ASC generation *in vivo* (11, 12) as well as ZIKV infection in humans (26, 27) or macaques (28), and the vaccination with YFV 17DD does not activate the same phenomenon (11). Considering that a direct correlation between kynurenine levels in the supernatants and the number of functional IgG^+^ ASC or secreted IgG titers were found for DENV4 TVP (Figures 5F,G), we evaluated whether other flavivirus would display it as well. Among all flaviviruses tested in this *in vitro* model, only the DENV4 LRV13/422 presented a similar correlation (*r* = 0.7395; *p* < 0.05) (Figures 6B–E). Therefore, the direct correlation between the kynurenine levels and functional IgG+ ASCs was only pointed out for DENV particles, which have been strongly linked to the ASC responses seen in human infections.

## Discussion

Although it is well-established that DENV infection causes a massive and transient DENV-specific ASC response during the disease symptomatology in patients (11, 12), the mechanisms related to this cellular process are still poorly understood. *In vivo*, the major DENV targets are monocytic cells (29). Among them, CD14+ CD16+ monocytes were described as capable to stimulate the B cell differentiation into ASCs *in vitro* (30). B cells are also susceptible to the DENV invasion (25), and infected cells have been found in the blood of Dengue patients (3). If B cells need to be infected to initiate that enormous ASC response, it has not been elucidated yet.

Correa and colleagues sought to clarify the DENV infection-mediated ASC differentiation through an *in vitro* strategy in which PBMCs from healthy donors were incubated with DENV particles. Only indirect suppositions of cellular responses could be taken from that study, though, as all measurements were based on the antibody secretion detected from culture supernatants. Whenever “cellular data” were analysed in that regard, purified B cells were selected for the incubation with DENV particles (25). However, our data showed that the functional ASC differentiation does not occur under that B-cell culture setting (Figure 3). On the other hand, purified B cells could clearly differentiate into ASCs upon incubation with mitogens (Figure 3) as previously demonstrated (21). It is known that the B cell differentiation into ASCs can be facilitated by the activation of receptors related to the innate immunity, such as Toll-like receptors (TLR) 3, 4, 7/8, and 9 (31). If viable DENV particles do not contain sufficient amounts of those TLR ligands in their composition, it has to be defined. Whereas the DENV NS1 protein was described to activate immune cells through TLR4 (32, 33), the DENV NS4 seems to be related to autophagy (34), which also has been associated with the ASC differentiation (35). More specifically, the DENV NS4 seems to interact with TAX1BP1 (36), restricting the *Blimp1* expression (37) that is critical for the B cell differentiation into ASCs. Of note, TAXBP1 KO mice present lower numbers of germinal centers in lymphoid organs as well as titers of antigen-specific antibodies (37).

Herein, we investigated the cellular basis of the DENV-mediated ASC generation using the same *in vitro* model previously described (25). We demonstrated an increased cell frequency with the ASC phenotype and function upon the PBMC culture with DENV4 TVP particles (Figure 1). Although the ASC numbers and percentage can rise up in Dengue patients if a secondary or a more severe DENV infection is considered (11, 12), our *in vitro* cellular differentiation model has shown to be independent of pre-existing DENV immunity (Supplementary Figure 4). To date, the literature is ambiguous regarding whether this DENV-mediated ASC differentiation is or is not related to DENV-specific memory B cells (10, 38, 39). Furthermore, cell cultures prepared with inactivated DENV particles did not allow the phenotypical and functional ASC differentiation (Figure 2) as reported *in vitro* and *in vivo* (25, 40, 41). In order to be differentiated into ASCs, our data also pointed out that B cells must have physical contact with the remaining PBMCs in the presence of DENV particles (Figure 4). In this context, a co-culture between B cells and CD14+ dermal monocytic cells presented higher frequency of ASCs and augmented antibody secretion *in vitro*. Whereas, the addition of TLR ligands to the co-cultures enhanced the B cell ability to differentiate into ASCs, the lack of physical contact among those cells diminished the ASC frequency and antibody production. In another co-culture experiment between B cells and CD14^+^ CD16^+^ monocytes, the ASC differentiation was dependent on the expression of BAFF, APRIL, and IL-10 (30).

Since viral loads are fundamental in the ASC differentiation process for infections, such as SIV (Silveira et al., unpublished data) and HIV (42), we wondered the DENV influence in our model. Neither viable DENV particles in the supernatants, nor viral transcripts were detected in the cells at day 7 of culture (data not shown). Since DENV-infected B cells have been found up to day 5 of cultures (5), earlier sample collection would potentially aid to define the role of DENV loads in our model. Recently, a model of human DENV infection presented a direct correlation between viremia and the magnitude of ASC response (29). However, other technologies would need to be applied to define the source of the DENV load for this B cell differentiation: monocytes or lymphocytes. Currently, DENV-infected cells are identified based on invasive methods, such as cell permeabilisation or nucleic acid isolation. These strategies inhibit the cell tracking to answer questions like “Can a DENV-infected B cell differentiate into an ASC? Are the B-cell viral loads associated to its ability to further differentiate into an ASC?” The use of an infective fluorochrome-labeled DENV clone may overcome those issues.

Multiple metabolic pathways have been associated to the ASC differentiation, such as glycolysis, oxidative phosphorylation, glucose uptake, inositol phosphate, and sterol metabolisms (43, 44). Furthermore, the DENV infection can stimulate the activation of the tryptophan metabolism. An elevated kynurenine concentration, the major product resultant from the tryptophan consumption, has been detected in the serum of DENV- and HIV-infected patients (7, 45, 46). This metabolite has been associated with a tolerizating immune response, especially for T cells (47–49). It does not seem to be the case for DENV, HIV, and SIV infections since they enhance the immunological status of patients (50–54). Another common characteristic of these viral infections is the induction of hypergammaglobulinemia (55–59), a hallmark of B cell activity that augments the ASC frequencies (29, 60, 61). The major difference between these scenarios is the target choice. Whereas a potent IgG^+^ ASC response is driven to the viral envelope during Dengue (11), the number of HIV-specific IgG^+^ ASCs display a low percentage of the total counterpart (60).

B cell responses have had their tryptophan metabolism evaluated in different models. The Zostavax booster vaccination elicited the expression of tryptophan metabolism-related genes, which were closely connected to genes linked to the ASC response (7). Among the relevant genes derived from this metabolic pathway are *IDO1* or *IDO2* (61). In addition, immunised IDO1 KO mice displayed greater humoral responses than controls with T-cell-independent antigens due to their higher capacity to proliferate and secrete antibodies (62). In a murine model of *Citrobacter rodentium*-induced colitis, IDO-deficient mice also displayed higher titers of IgA and IgG than controls (63). Genes with expressions tightly bound to the IDO activity, such as eukaryotic initiation factor 2 alpha (*eIF2alpha*) and kinase general control non-derepressible-2 (*GCN2*), can enhance the *BLIMP1* (or *PRDM1*) expression in a tryptophan-depleted condition. On the other hand, the BLIMP1 protein binds to the IDO promoter, repressing its expression (64). The tryptophan degradation also modifies the expression of *SLC7A5* (65) and *WARS* during this process in cancer cells (66) and ASCs (67). Regarding cancer cells, the prognostic of diffuse large B-cell lymphoma, mainly composed by ASCs, and its outcome for a patients have been strongly connected to the *IDO* expression and the presence of determined serum kynurenine concentrations (68–71). The clinical progression of multiple myeloma, also characterised by transformed long-lived ASCs, has also been connected to the IDO activity (72). In our model, while no tryptophan degradation was identified in the mitogen-driven ASC differentiation, a strong activation of this amino acid metabolism seemed to occur during the DENV-mediated ASC generation *in vitro*. Either a lab-adapted or a clinical DENV isolate stimulated the tryptophan consumption, accumulating kynurenine in the supernatants of the PBMC-DENV cultures. Overall, an increased expression of *IDO1* and *IDO2* transcripts was noticed in those cell cultures. More importantly, the tryptophan and kynurenine concentrations were directly correlated to the magnitude of an DENV-specific ASC response *in vitro* (Figure 5). Downstream metabolites were also detected in higher amounts in the PBMC-DENV cultures than in controls (Supplementary Figure 4). In corroboration with these data, we also observed increased amounts of transcripts related to the ASC survival or differentiation, such as *TNFS13B* and *IL-10* (Supplementary Figure 5) as previously described (30, 73–75). Thus, it is suggested that the tryptophan degradation would be the cause and not consequence of the DENV-mediated ASC differentiation *in vitro*, as it seems to occur earlier than the BLIMP1 expression (64). The role of this metabolic pathway in the massive ASC response detected in Dengue patients is still to be ascertained.

Other flaviviruses present lower potential to induce robust ASC responses *in vivo*, as the DENV does. Among them, we tested two ZIKV strains (26–28) and the attenuated YFV 17DD (11) in our model. Their ASC data obtained *in vitro* reinforced the same scenario observed *in vivo* (Figure 6A). In terms of magnitude, ZIKV or YFV 17DD-treated PBMC cultures had lower median kynurenine concentrations than those treated with DENV particles. Moreover, the cultures with ZIKV or YFV 17DD showed no correlation between their kynurenine concentrations and the respective ASC magnitudes (Figures 6B–D). It is likely that relevant factors for the ASC differentiation, such as BAFF, APRIL, or IL-10 were reduced in those flavivirus-PBMC cultures in comparison to the DENV counterpart. Thus, our data suggests that the activation of the tryptophan metabolism as a biomarker of the DENV-mediated ASC responses *in vivo*.

## Conclusions

The *in vitro* model of B cell differentiation into ASCs has provided relevant evidence on how this phenomenon operates. Indeed, the presence of mitogens or DENV particles in the PBMC cultures did elicit the ASC differentiation. Regarding the viral participation in this cell differentiation process, it requires viable particles rather than inactivated forms in order to stimulate the acquisition of the ASC phenotype and function. Moreover, those PBMC-containing B cells requested the cell–cell contact with the remaining PBMCs to fully differentiate into ASCs in the presence of DENV particles. In contrast to the mitogen-mediated ASC differentiation, we demonstrated that both laboratory-adapted and clinically isolated DENV particles can activate the tryptophan metabolism in PBMC cultures in a magnitude directly correlated with the generated ASC numbers. Flaviviruses known to stimulate inferior ASC responses *in vivo*, such as Zika and the attenuated Yellow Fever viruses, presented no correlation between their abilities to stimulate tryptophan degradation and their PBMC-derived ASC differentiation *in vitro*. Other conditions capable of triggering this amino acid degradation pathway, such as HIV and SIV infection, B cell lymphoma, and multiple myeloma, show similarities with the DENV-mediated B cell differentiation. Thus, the activation status of the tryptophan metabolism may affect the B cell fate during an infectious disease by stimulating or not the ASC differentiation. Furthermore, such information may help researchers to understand why specific adjuvants stimulate stronger humoral responses than others in vaccine formulations. Eventually, strategies based on the effects of the tryptophan metabolism may be applied to improve B cell responses against other pathogens or diseases.

## Data Availability Statement

All datasets generated for this study are included in the article/Supplementary Material.

## Ethics Statement

All blood samples evaluated in this study were voluntarily donated by healthy adults from the University of São Paulo and Instituto Carlos Chagas/Fiocruz-PR (ICC/Fiocruz-PR) (Brazil). All patients provided written informed consent prior to the study enrollment. Experimental protocols and procedures were reviewed and approved by the Institutional Ethics Committee (CAEE 68875417.9.0000.0067) regulated by the Conselho Nacional de Ética em Pesquisa.

## Author Contributions

VB conducted the execution of all experiments, except the ELISA, and contributed to the statistical analyses, review, and interpretation of data sets. AHDC contributed to the experiment execution, review, and interpretation of data sets. MB and MS generated proteomics data. PG-D provided bioinformatics support. SP conducted the ELISA. LF provided the ELISA kit and contributed to the experiment execution, review, and interpretation of data sets. HN provided bioinformatics data and support, and contributed to review and interpretation of data sets. AC contributed to the generation of proteomic data, review, and interpretation of data sets. PW provided viral strains and contributed to the experiment execution, generation of samples, review, and interpretation of data sets. ES conceptualised and directed the project, provided the statistical analyses, interpreted the data sets, and prepared the manuscript with input from all authors.

### Conflict of Interest

The authors declare that the research was conducted in the absence of any commercial or financial relationships that could be construed as a potential conflict of interest.
