# Supplementary figures and images for: Flavivirus-Mediating B Cell Differentiation Into Antibody-Secreting Cells in Humans Is Associated With the Activation of the Tryptophan Metabolism
Source: Front Immunol. 2020 Feb 11;11:20. doi: 10.3389/fimmu.2020.00020 (PMC7026258; doi:10.3389/fimmu.2020.00020)

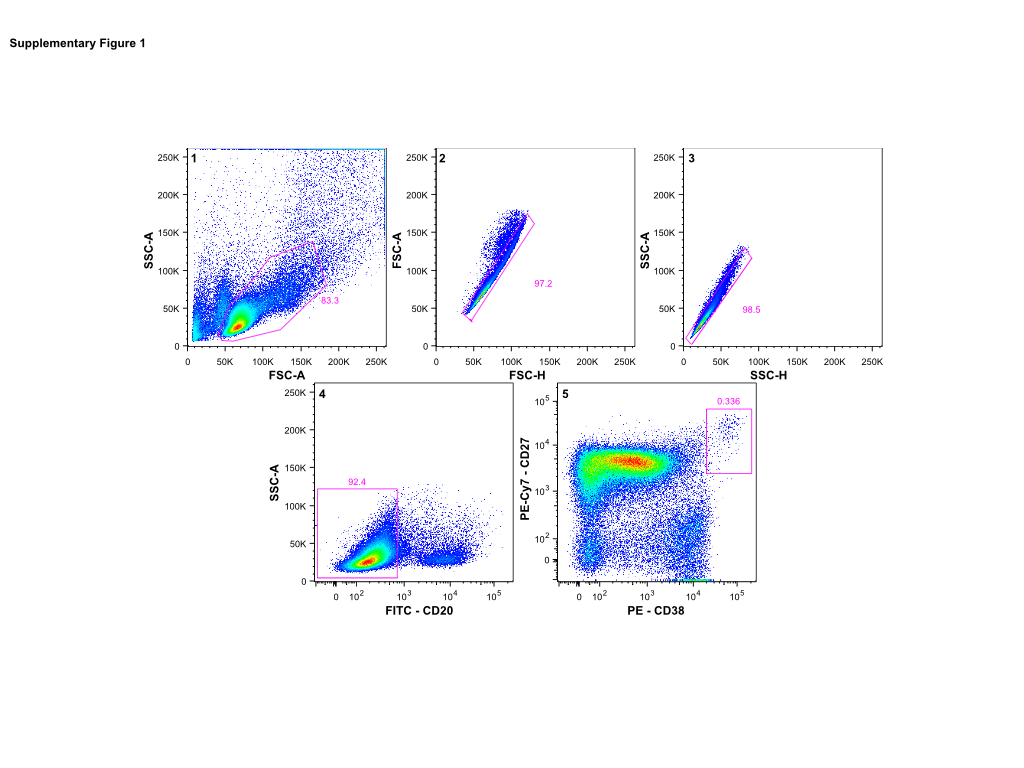

Supplement: Supplementary file 1 [file Image_1.TIFF]

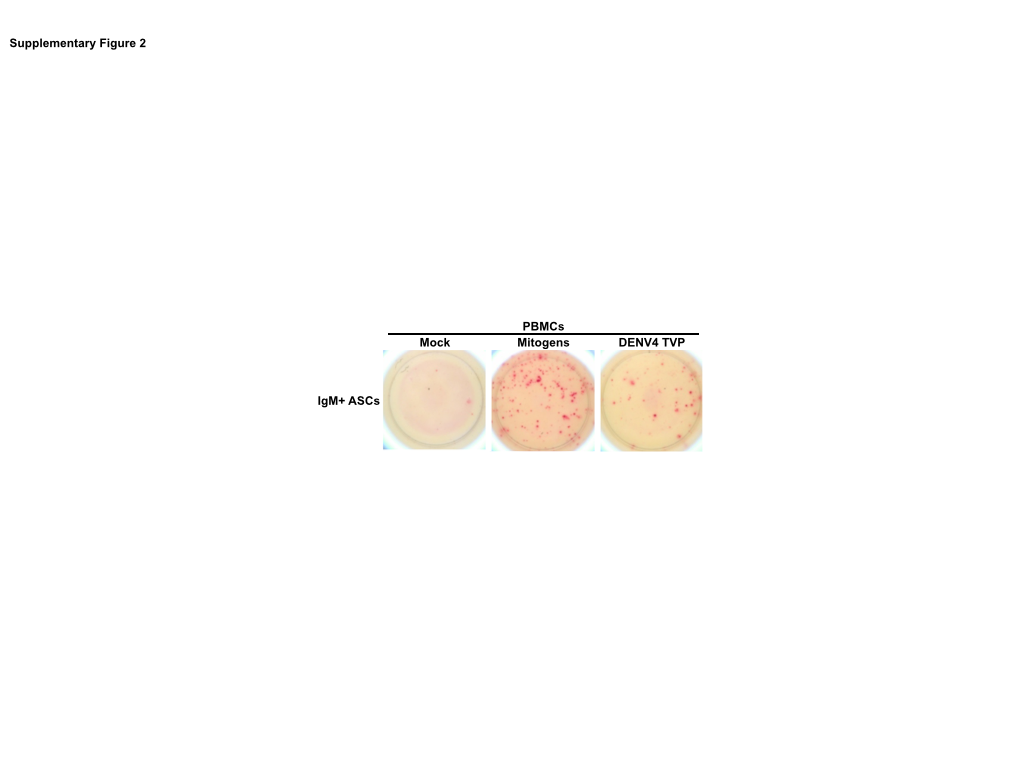

Supplement: Supplementary file 2 [file Image_2.TIFF]

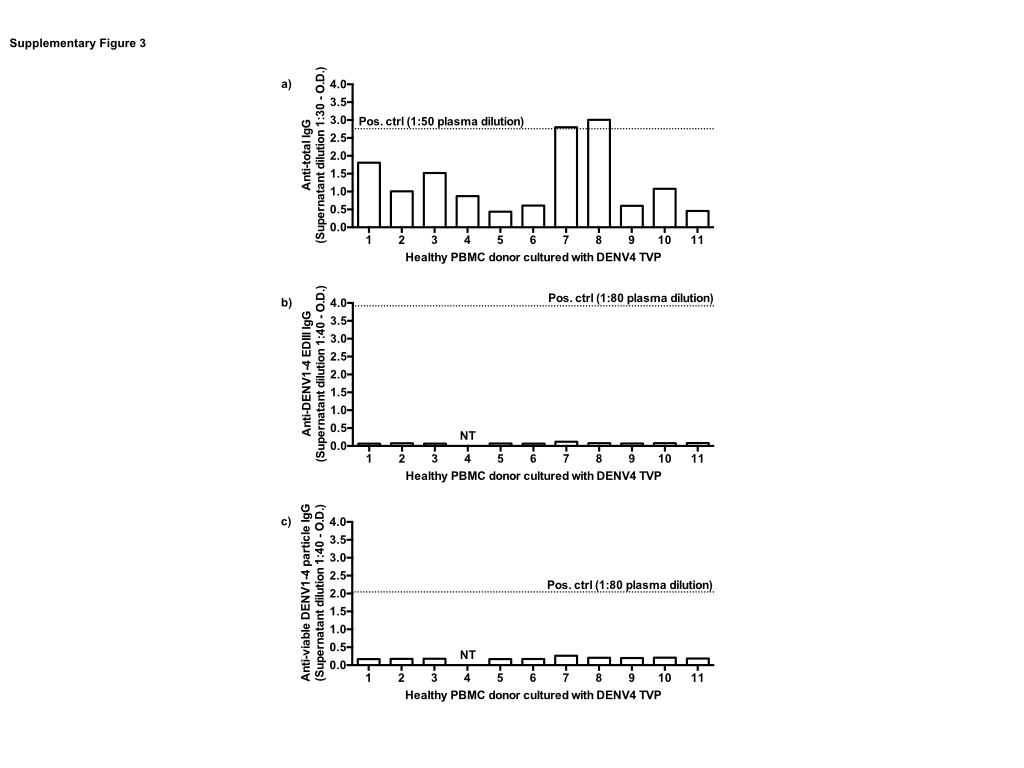

Supplement: Supplementary file 3 [file Image_3.TIFF]

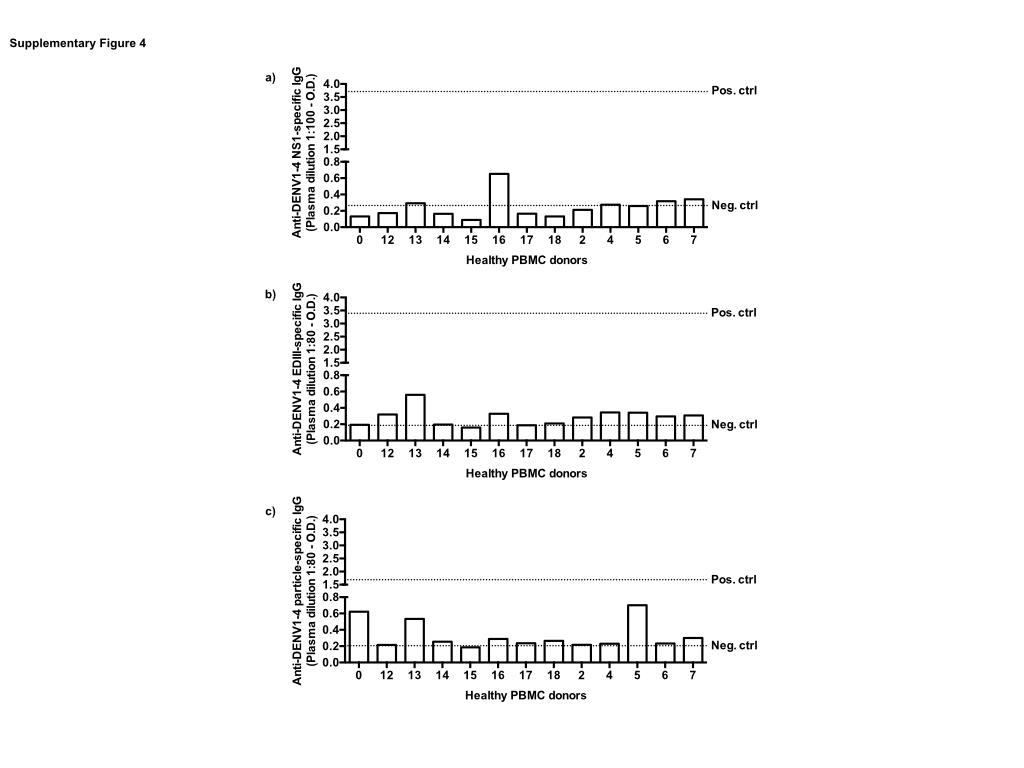

Supplement: Supplementary file 4 [file Image_4.TIFF]

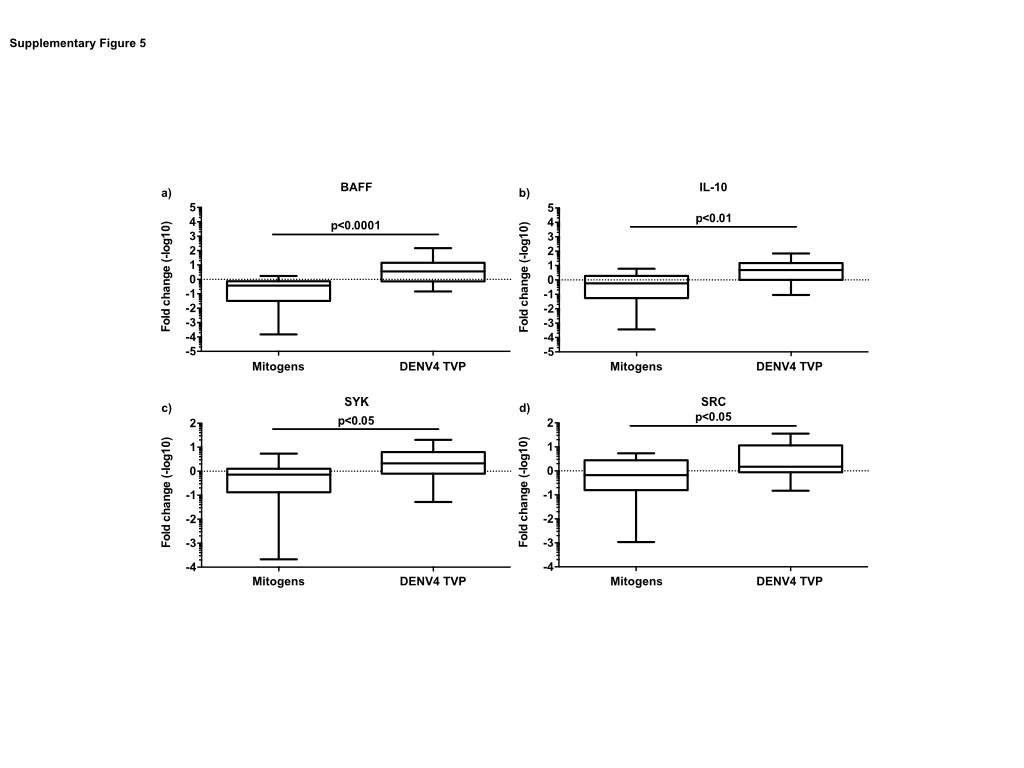

Supplement: Supplementary file 5 [file Image_5.TIFF]

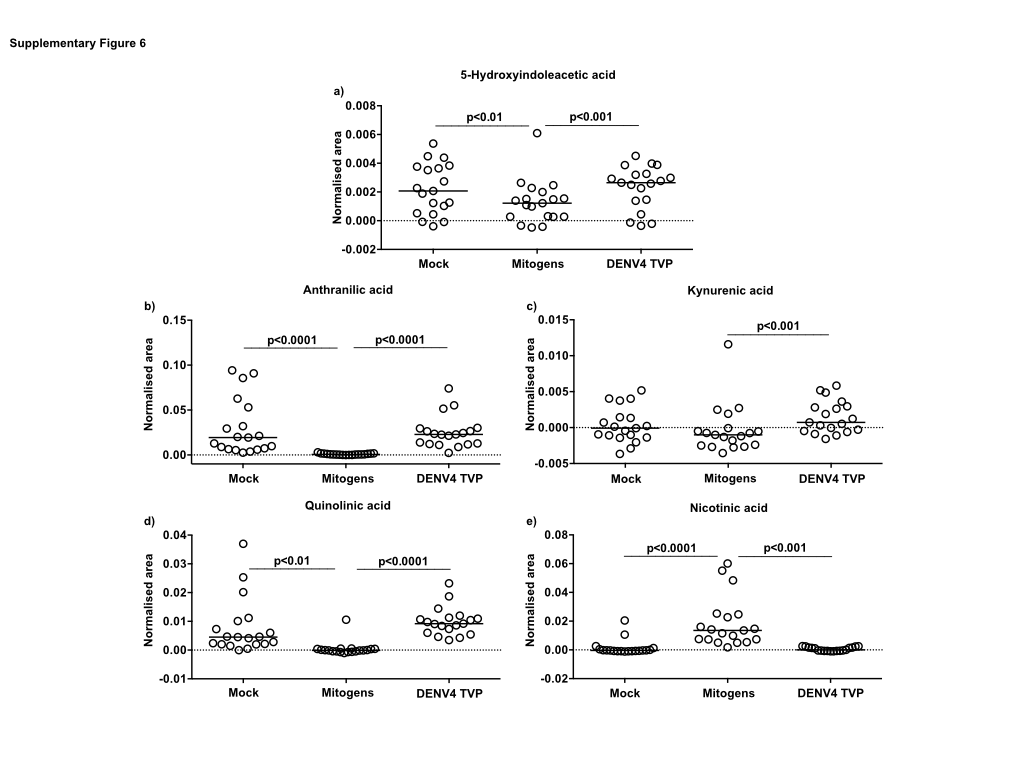

Supplement: Supplementary file 6 [file Image_6.TIFF]
