# Supplementary material for: Flavivirus-Mediating B Cell Differentiation Into Antibody-Secreting Cells in Humans Is Associated With the Activation of the Tryptophan Metabolism
Source: Front Immunol. 2020 Feb 11;11:20. doi: 10.3389/fimmu.2020.00020 (PMC7026258; doi:10.3389/fimmu.2020.00020)
Supplement: Supplementary file 7 [file Data_Sheet_1.docx]

Supplementary figure legends

**Supplementary Figure 1 -** Sequential gating strategy to determine the human antibody-secreting cell (ASC) phenotype. PBMCs were cultivated with DENV4 TVP particles in the MOI of 10 for 7 days. Cells were harvested, and labelled for surface staining antigens with fluorochrome-conjugated antibodies. A) The representative FACS data showing an enrichment for ASCs: 1) extended lymphocyte including blasting cells; 2–3) singlets; 4) CD20− cells; 5) CD38hi CD27hi cells.

**Supplementary Figure 2 -** Dengue virus triggers the IgM secretion upon culture with PBMC-containing B cells. A total of 3.3 x 104 cultured PBMCs were harvested at day 7 of culture and added per well of ELISPOT plates to enumerate IgM-secreting cells in each culture condition.

**Supplementary Figure 3 -** IgG secretion detected in the supernatants of PBMC-DENV cultures did not react against DENV1-4 EDIII recombinant proteins or viable DENV1-4 particles. Reactivity of supernatant samples harvested at day 7 of cultures were tested through ELISA in different dilutions: a) 1:30 - human IgG; b) 1:40 - EDIII recombinant proteins from DENV1-4 serotypes; c) 1:40 - viable DENV particles from DENV1-4 serotypes. NT - not tested.

**Supplementary Figure 4 -** Small IgG reactivity of plasma samples from blood donors of this study against distinct DENV targets through ELISA. Plasma samples were tested in different dilutions: a) 1:100 - NS1 recombinant proteins from all 4 serotypes; b) 1:80 - EDIII recombinant proteins from DENV1-4 serotypes; c) 1:80 - DENV viable particles from DENV1-4 serotypes.

**Supplementary Figure 5 -** Differential gene expression profile (fold change) between PBMC cultures stimulated with DENV4 TVP, Mitogens, or Mock supernatants for genes related to cellular signalling and B cell differentiation into ASCs. Targeted genes were: a) BAFF; b) IL-10; c) SYK; d) SRC. Data are represented as boxes and whiskers (10-90% percentile). Dashed lines represents the gene expression within the Mock culture condition.

**Supplementary Figure 6 -** Activation of the tryptophan metabolism through either serotonine (a) and kynurenine pathways (b-e) in the in vitro model of DENV-mediated ASC differentiation in comparison to mitogen-stimulated culture. Different PBMC cultures derived from 19 healthy donors had their supernatants obtained at day seven of the culture for the measurement of the following metabolites: a) 5-hydroxyindoleacetic acid; b) Anthranilic acid; c) Kynureninic acid; d) Quinolinic acid; e) Nicotinic acid. Dashed lines represent the standard amount of the metabolite found in the culture medium kept under the same conditions, but without cells.

Supplementary table legends

Supplementary Table 1 - Fluorochrome-conjugated antibodies used to estimate the percentage and absolute number of antibody-secreting cells within the cell cultures.

| Human antigen | Clone | Fluorophore | Supplier |
| --- | --- | --- | --- |
| CD20 | 2H7 | FITC | Biolegend |
| CD38 | HI72 | PE-Cy7 | Biolegend |
| CD27 | CD27M-t271 | PE | Biolegend |

Supplementary Table 2 - qRT-PCR primers.

| Target | Forward primer  5’-3’ | Reverse primer  5’-3’ | Amplicon size (bp) | Reference |
| --- | --- | --- | --- | --- |
| *SYK* | AAAGACAAATGGAAAGTTCCTGA | CTTTGTCGATGCGATAGTGC | 104 | (Grammatikos et al., 2013) |
| *IL10* | GGTTGCCAAGCCTTGTCTGA | AGGGAGTTCACATGCGCCT | 101 | (Pfaffl, 2001) |
| *SRC* | AGTGCTGGCGGAAGGAGCCT | ATCCAAGCCGAGAAGCCGGT | 149 | (Laghi & Al, 2001) |
| BAFF  (*TNFSF13B)* | AGGCAACTCCAGTCAGAACAGC | TCATCCCCAAAGACATGGACC | 303 | (Schaumann et al., 2007) |
| IDO1 | AGACCACAAGTCACAGCGCC | TTGGCAAGACCTTACGGACAT | 70 | (Opitz et al., 2011) |
| IDO2 | TGCTTCATGCCTTTGATGAG | GAAGGCCTTATGGGAAGGAG | 104 | (Opitz et al., 2011) |
| DENV | GACTAGTGGTTAGAGGAGACC | GTCTCCTCTAACCTCTAGTCCT | 155 | Laboratório de Virologia Molecular, Instituto Carlos Chagas, ICC/Fiocruz-PR, Curitiba, PR - Brazil |

Supplementary references

GRAMMATIKOS, A. P. et al. Spleen Tyrosine Kinase (Syk) Regulates Systemic Lupus Erythematosus (SLE) T Cell Signaling. PLoS ONE, v. 8, n. 8, 27 ago. 2013.

LAGHI, L. et al. Lack of mutation at codon 531 of SRC in advanced colorectal cancers from Italian patients. British Journal of Cancer, v. 84, n. 2, p. 196–198, 2001.

OPITZ, C. A. et al. The indoleamine-2,3-dioxygenase (IDO) inhibitor 1-methyl-d-tryptophan upregulates IDO1 in human cancer cells. PLoS ONE, v. 6, n. 5, 2011.

PFAFFL, M. W. A new mathematical model for relative quantification in real-time RT-PCR. Nucleic Acids Research, v. 29, n. 9, p. 45e – 45, 1 maio 2001.

SCHAUMANN, D. H. S. et al. VCAM-1-positive stromal cells from human bone marrow producing cytokines for B lineage progenitors and for plasma cells: SDF-1, flt3L, and BAFF. Molecular Immunology, v. 44, n. 7, p. 1606–1612, 2007.
